# Insurance Expansion During Pregnancy

**DOI:** 10.1002/hec.4978

**Published:** 2025-05-26

**Authors:** Philip Hochuli, Christian P. R. Schmid

**Affiliations:** ^1^ CSS Lucerne Switzerland; ^2^ CSS Institute Lucerne Switzerland

**Keywords:** cost‐sharing exemption, demand for health, health insurance, pregnancy care and maternal health

## Abstract

We analyze how the abolition of cost‐sharing in health insurance affects pregnant women's gross spending on health care services using an exogenous policy change in Switzerland. Using non‐linear regression, we find that the policy slightly increases average gross spending, contrasting policymaker expectations of no impact on demand. More importantly, however, we find strong demand responses for specific types of services (physiotherapy, laboratory services), in particular for below‐median income individuals. Within this group, we find that physiotherapy increases as much as 50% in response to the policy change. Additionally, we find suggestive evidence of a relative improvement of newborn health among individuals with below‐median income, indicating that additional use of healthcare services may be beneficial. However, we find no evidence of an impact on maternal health. These results highlight that cost‐sharing policies—such as the one we examine—need to balance trade‐offs between reducing healthcare costs and addressing the health and equity implications of such policies.

## 1 Introduction

Health insurance involves a fundamental trade‐off between the gains from risk reduction and the welfare loss from the individuals' incentive to demand more health care when insured (Arrow 1963; Zeckhauser 1970; Spence and Zeckhauser 1971; Pauly 1974). Public as well as private insurers therefore rely on patient cost‐sharing including deductibles, co‐insurance and co‐payments to reduce over‐consumption of care. While patient cost‐sharing indeed decreases health care expenditures (Newhouse 1993; Finkelstein et al. 2012; Aron‐Dine et al. 2013), it seems also to have unintended consequences. Recent empirical research suggests that patients, in response to an increase in cost‐sharing, reduce care where the benefits would outweigh the costs (Chandra et al. 2010; Choudhry et al. 2011; Brot‐Goldberg et al. 2017). As a result, cost‐sharing can have negative health consequences which should be taken into account when designing health plans (Chandra et al. 2021).

In this paper, we study the effects of a cost‐sharing *exemption* for pregnant women on their demand for health care in the context of compulsory insurance in Switzerland. The exemption has been driven by the desire to prevent women with pregnancy complications from paying more than women without any complications during pregnancy. In addition, the intention was to prevent pregnant women from not seeking necessary care because of cost‐sharing.^1^ Since the implementation of the exemption on March 1st, 2014, *all* health care services have been exempt from cost‐sharing between the beginning of the 13th week of pregnancy and the end of the 8th week after delivery.^2^ In other words, pregnant women have free health care in this limited period. In contrast, cost‐sharing for the first 12 weeks of pregnancy has remained the same. As a result, we can compare pregnancy weeks before and after the policy introduction as well as pregnancy weeks with different cost‐sharing within ongoing pregnancies. This quasi‐experimental setting allows us to identify the effect of a cost‐sharing exemption on the expectant mothers' demand for health care. In addition, we can consider the subsequent effect on maternal health and the newborns' health.

Using data from a large Swiss health insurer, we apply a difference‐in‐differences‐type regression model to estimate the effect of the cost‐sharing exemption. While most services do not exhibit a demand response, we find an increase in the demand for physiotherapy (physical therapy) by 30% and laboratory by 5% (or approx. 0.4 and 1.1 Swiss Francs per pregnancy week, respectively). A heterogeneity analysis by income reveals that these effects are driven by pregnant women with below‐median income. In this group, our estimates suggest that overall health care spending increased by approx. 4.9% (or 4.4 Swiss Francs per pregnancy week) in response to the cost‐sharing exemption. We estimate the overall demand response to be around 6 to 7 million Swiss Francs per year. While our estimates indicate increased utilization, we cannot fully rule out that some of the observed demand response reflects a shift in the timing of care (demand shifting) rather than an overall increase in the volume of care. In addition, however, the cost‐sharing exemption shifted between 40 and 50 million Swiss Francs of spending from individuals to health insurers per year. Turning to health, we have a limited set of health indicators on both the mother (inpatient stay post pregnancy, inpatient psychiatric stay post pregnancy, drug‐based chronic disease indicators) and the child (low birth weight indicator, DRG case weight). While we do not find evidence of a health impact of the policy on the mother, our analysis suggests a small positive health impact on newborns of below‐median income individuals, in line with the positive demand response.

Our study complements the existing evidence on insurance coverage (expansion) for pregnant women and the effects on prenatal care, maternal health and child health (see e.g., Currie and Gruber 1996a, 1996b; Currie and Grogger 2002; Conway and Deb 2005; Conway and Kutinova 2006; Guldi and Hamersma 2023). Based on Medicaid, these studies find increases in the use of prenatal care, improved health of the mother, and—to a limited extent—also better infant health including lower infant mortality. We contribute to this literature analyzing the effects of a cost‐sharing exemption in the context of comprehensive, mandatory health insurance in a regulated competition setting. Consequently, our results are based on a population with a broader socio‐economic background compared to Medicare enrollees and can also be informative for health care systems with mandatory insurance. In addition, we complement a recent study by Epure et al. (2023) focusing on the health impact of the same reform. Using the Swiss birth register, they find a small positive effect on newborn health which is more pronounced among individuals not at risk of poverty. Thus, their primary focus lies on newborn health while our paper centers on healthcare spending. In addition, we also provide some evidence on maternal health. We furthermore contribute to the literature on the effects of patient cost‐sharing on the demand for health care in general (see e.g., Cutler and Zeckhauser 2000; McGuire 2012, for an overview) and in Switzerland (see e.g., Boes and Gerfin 2015; Gerfin et al. 2015). In principle, a reduction in patient cost‐sharing leads to more demand for health care. Our results corroborate this finding, however, the estimated demand response is limited to specific services in our case.

The remainder of this article is structured as follows: In Section 2 we provide the institutional background on the health insurance market in Switzerland and the policy setup. In Section 3 we first introduce our estimation strategy followed by an overview of the data for our research. In Section 4 we provide regression results including robustness tests. We end with a summary and conclusions in Section 5.

## 2 Institutional Background

Similar to Germany and the Netherlands, compulsory health insurance in Switzerland is based on principles of regulated competition (the following description draws on Schmid et al. 2018). Insurers and health care providers compete on price and quality while regulation ensures risk solidarity, individual affordability, accessibility of health plans, and access to care. Health plans are offered by approximately 60 private insurers that are obliged to accept all individuals who wish to enroll (open enrollment). Health insurers must offer the standard health plan, which includes free choice of physician, an individual deductible of CHF 300 and a co‐insurance rate of 10% up to the stop‐loss amount of CHF 700. In principle, insurers have to charge community‐rated premiums that vary only with region and three age categories (children, young adults aged 19–25, and adults). However, individuals can opt for higher deductibles ranging from CHF 500 to 2500 and managed care options including telemedicine, preferred provider, and health maintenance organization health plans.^3^ Opting for a higher deductible and/or a managed care option results in a premium reduction, but is strongly regulated to maintain risk solidarity.

Each health plan has to offer the same, rather comprehensive coverage. In case of sickness, outpatient and inpatient services performed by physicians are covered as well as physician‐directed services performed by other healthcare providers including pharmacists (e.g., prescription drugs), physiotherapists, psychotherapists, laboratories, and so on.^4^ In case of pregnancy, the coverage additionally includes services provided by midwifes and birth houses. Note that health plans must not extend the coverage, but health insurers are allowed to sell supplementary health insurance plans that cover additional services (e.g., dental care). Regarding provider payment, outpatient services are reimbursed on a fee‐for‐service base, which is negotiated separately for physicians, midwifes, laboratories, and so on. In contrast, reimbursement of somatic and maternity‐related inpatient care is based on diagnosis related groups (DRG) since the beginning of 2012 (for details, see Hochuli 2020).

**FIGURE 1:**
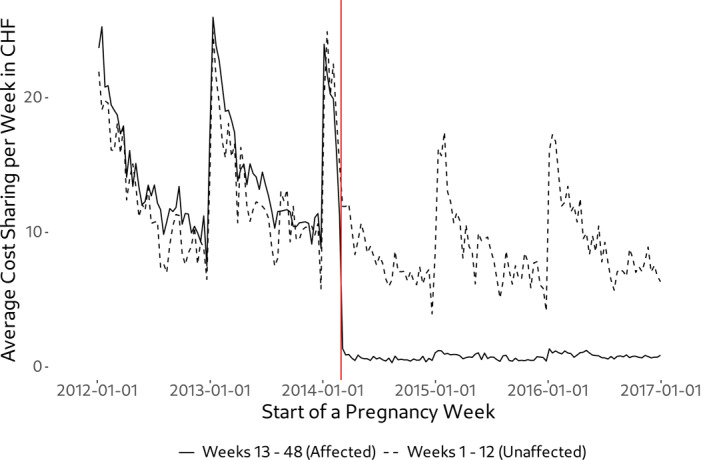
Effect of the policy change on weekly cost‐sharing. The graph shows average weekly cost‐sharing of (expectant) mothers between the 1st and 12th week of pregnancy (dashed line) as well as between the 13th week of pregnancy and the 8th week after delivery (solid line). Starting March 1st in 2014 (red vertical line), all health care use of (expectant) mothers within the 13th week of pregnancy and the 8th week after delivery has been exempt from patient cost‐sharing. As a result, weekly cost‐sharing drops to zero after this date.

**FIGURE 2:**
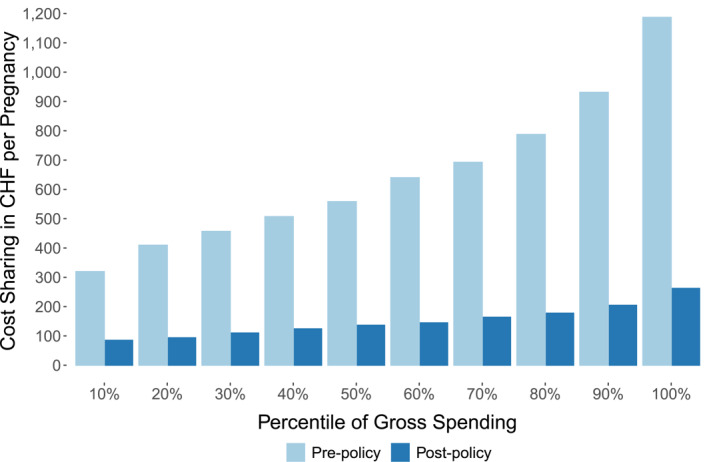
Average cost‐sharing during pregnancy (week 1 until 8th week after delivery) in chf, by decile of total gross spending of the pregnancy. The figure categorizes pregnancies into deciles of spending. For each decile, the figure then shows the average cost sharing of those pregnancies over the entire course of the pregnancy (week 1 until week 8 after delivery) in both the pre‐policy and post‐policy period. Pregnancies in year 2014 have been excluded.

While all these services are generally subject to cost‐sharing, health care is nowadays free for pregnant women between the 13th week of pregnancy and the 8th week after delivery.^5^ However, before March 1st, 2014, pregnancy‐related health care including the treatment of pregnancy complications had been subject to patient cost‐sharing. In order to prevent women with pregnancy complications from paying more than women without any complications, the federal parliament decided in June 2013 to *completely* eliminate cost‐sharing during maternity starting from the 13th week of pregnancy.^6^ Consequently, and as illustrated in Figure 1, pregnant women's out‐of‐pocket price for healthcare is zero after the 12th week of pregnancy since the policy change. However, Figure 1 also reveals that cost‐sharing until the 12th week of pregnancy barely changes, which in turn implies that the out‐of‐pocket price in these weeks was constant over time.^7^ In Figure 2 we show that the policy change achieved its intended consequence in the sense that more costly pregnancies, and thus pregnancies with complications, benefited much more from the policy than low‐cost pregnancies. Among the top‐percentile of pregnancies in terms of health care expenditures, average cost sharing dropped from approx. CHF 1200 in the pre‐policy period to approx. CHF 250 in the post‐policy period. In consequence, differences in out‐of‐pocket expenditures over the course of a pregnancy strongly declined.

## 3 Empirical Approach

### 3.1 Estimation Strategy

Given the policy change at hand, we employ a difference‐in‐differences type framework for estimating its effect on health care spending. However, as the cost‐sharing exemption applies to weeks of pregnancy and not individuals, we abstain from using the standard terminology. In particular, we refer to the weeks after the 12th week of pregnancy as *affected* weeks while the weeks 1–12 are referred to as *unaffected* weeks. This classification of pregnancy weeks is analogous to the distinction between the treatment and the control group in the standard difference‐in‐differences framework and thus time‐independent. Basically, we compare the difference in health care demand of affected and unaffected weeks before the cost‐sharing exemption came into effect with the corresponding difference after the policy change. In order to identify the causal effect, we rely on the following common trend assumption: We assume that, in absence of the policy change, health care spending in the affected weeks would have evolved the same way as health care spending did evolve in the unaffected weeks. We discuss the validity of this assumption in more detail when describing our data.

In our empirical approach, we have to take into account two key features of health care cost data. First, health care costs rise over time and exhibit a quite constant growth rate. Second, the health care cost distribution is zero‐censored and positively skewed. Therefore, we follow Boes and Gerfin (2015) by modeling the expected spending using a generalized linear model with log‐link and quasi‐poisson error distribution (adjusting for overdispersion). In other words, we assume that the logarithm of expected gross spending is a linear function of explanatory variables and that errors follow a (quasi‐)poisson distribution, conditional on the mean. Consequently, estimated coefficients can be interpreted as approximate percentage changes in the dependent variable.

Equation (1) shows our specification where i denotes the pregnancy case, w denotes the pregnancy week, and $C_{iw}$ denotes weekly gross spending. Further, $\tau_{i}$ denotes year fixed effects based on the year of delivery, which controls for general cost growth, and $D_{w}$ is a dummy variable taking the value of 1 if the pregnancy week belongs to the affected group, which corresponds to the “treatment indicator” in the difference‐in‐differences analogy. The coefficient $\beta_{1}$ therefore denotes the fixed effect for the affected pregnancy weeks, that is, it controls for structural, time‐invariant differences between the affected and the unaffected pregnancy weeks. Finally, the sum expression denotes interaction terms of the year of delivery $\left(T_{i}\right)$ with our dummy variable for the affected group $\left(D_{w}\right)$, and $\gamma_{T}$ then denotes the year‐specific coefficient on these interaction terms. In our analysis, the year prior to the policy change serves as reference year, which is the year 2013. We estimate our model with standard errors clustered at the pregnancy case level.

(1) $$E\left[C_{iw}|\tau_{i},D_{w},T_{i}\right]=\exp\left(\tau_{i}+\beta_{1}D_{w}+\sum_{T\neq 2013}\gamma_{T}\left(T_{i}\times D_{w}\right)\right)$$

In analogy to the standard difference‐in‐differences setup, we are primarily interested in coefficients $\gamma_{T}$, which capture the year‐specific causal effect of the policy change on health care costs in the affected weeks. In presence of a policy impact, we expect to see positive and significant coefficients on these interactions for years ≥2015 (and possibly already in 2014). In addition, the coefficient $\gamma_{2012}$ needs to be close to zero and insignificantly different from zero as we expect a violation of parallel trends otherwise.

### 3.2 Data

For our analysis, we have access to all DRG reimbursed deliveries from one of Switzerland's largest health insurers (CSS) as well as detailed individual‐level data between 2012 (year of introduction of the national DRG system) and 2019, including the exact date of medical treatments and use of services. While we thus know the date of childbirth, we do not observe when the pregnancy started. Instead, we rely on the median gestation length (277 days, standard deviation 15 days) to infer the pregnancy start and to construct a data set on the level of the pregnancy week.^8^ Using the date of medical treatment, we can assign health care costs to pregnancy weeks. As a result, we have health care expenditures on various cost categories including outpatient care, inpatient care, laboratory services, physiotherapy, drugs, and medical equipment for each week of a pregnancy. In addition, we observe the (expectant) mothers' age, language, canton of residence, an income indicator, and their health plan in terms of the selected deductible.

In total, our initial data set consists of 107,555 unique pregnancies of women in reproductive age (i.e., aged 15–49 according to the World Health Organization), which corresponds to an annual average of 13,444 childbirths. Note that the total number of inpatient childbirths in Switzerland between 2012 and 2019 is 686,028 (Federal Statistical Office 2022). Given CSS’ average market share among women between age 19 and 50 in this period (15.7%), the observed 13,444 childbirths is close to the expected number of childbirths (13,463).^9^ Therefore, in terms of pregnancies, our data seems to be representative for Switzerland. As individuals can switch their health insurer on an annual basis, however, there are two additional aspects to consider. First, CSS has exhibited considerable growth in the number of insured since 2012, which is also reflected in strong growth in the number of pregnancies.^10^ Second, some pregnancies are not fully observed as some women switched to CSS or left CSS during an ongoing pregnancy. In our main analysis, we exclude these 21,167 pregnancies (≈20% of cases). Table A1 (in the Appendix) provides descriptive summary statistics for these cases. On average, these cases are somewhat younger (approx. 1 year), are less often German‐speaking (by approx. 5% points), and have a sligthly higher deductible (by approx. CHF 60). We check our analysis for robustness against exclusion of these cases.

Turning to health care expenditures, if the cost‐sharing exemption induces a demand response, the costs in the affected weeks increase relative to the costs in the unaffected weeks. However, as many services during pregnancy are non‐elective, we expect to see differences in the demand response between elective and non‐elective care. In Table 1, we summarize average weekly gross spending in CHF for various cost categories. In the bottom half of the table we also include cost categories that we do not consider for our analysis (e.g., midwife services, birth preparation but also inpatient care). Since those categories occur almost exclusively in the affected group they are inappropriate for a comparison of cost growth. Our variable “total” already adjusts for these cost categories by excluding them.

**TABLE 1:** Average weekly gross spending by cost category, group, and year.

Variable	Group	2012	2013	2014	2015	2016	2017	2018	2019
Drugs	Unaffected	6.9	6.6	7.4	6.4	7.3	7.9	8.8	8.1
	Affected	9.4	8.5	10.0	9.8	9.9	10.8	11.3	11.7
Equipment	Unaffected	0.6	0.7	0.8	0.7	0.8	0.7	0.8	0.7
	Affected	3.1	3.2	3.4	3.7	3.9	3.9	4.0	4.0
Laboratory	Unaffected	32.6	33.4	33.9	34.4	38.2	39.5	40.0	41.4
	Affected	21.7	22.0	22.1	23.3	25.7	27.4	28.9	28.7
Outpatient	Unaffected	42.5	43.2	45.1	46.7	48.1	49.4	49.5	52.1
	Affected	56.5	58.1	59.8	63.4	65.7	68.0	66.2	68.3
Physio	Unaffected	0.7	0.7	0.8	0.9	0.8	1.0	1.0	1.1
	Affected	1.2	1.3	1.5	1.8	2.1	2.4	2.6	2.9
Other	Unaffected	2.8	2.4	3.7	2.6	2.8	3.0	2.8	2.8
	Affected	4.5	4.5	4.6	4.5	4.0	4.5	4.5	4.3
Total	Unaffected	86.2	87.0	91.7	91.8	98.0	101.5	102.9	106.3
	Affected	96.3	97.6	101.4	106.3	111.2	116.9	117.4	119.8
Birth prep.	Unaffected	0.0	0.0	0.0	0.0	0.0	0.0	0.0	0.0
	Affected	0.7	0.7	0.7	0.8	1.0	1.1	1.2	1.3
Care	Unaffected	0.0	0.1	0.1	0.1	0.1	0.1	0.2	0.1
	Affected	0.3	0.4	0.7	0.7	0.9	1.0	1.2	1.2
Inpatient	Unaffected	4.6	3.6	4.0	3.5	3.8	3.3	3.3	3.5
	Affected	134.5	131.8	129.0	129.1	127.5	126.8	125.4	122.9
Midwife	Unaffected	0.4	0.3	0.4	0.3	0.3	0.2	0.1	0.2
	Affected	12.1	12.7	13.9	17.1	20.9	22.6	24.5	26.4
Nursing advice	Unaffected	0.0	0.0	0.0	0.0	0.0	0.0	0.0	0.0
	Affected	0.9	1.2	1.1	0.8	0.4	0.3	0.3	0.3
Vaccination	Unaffected	0.0	0.0	0.1	0.0	0.0	0.0	0.0	0.0
	Affected	0.0	0.0	0.1	0.0	0.2	0.3	0.4	0.7

*Note:* The table shows average weekly spending (in CHF, rounded to one digit) by year for different cost categories and separately for the affected and unaffected weeks of pregnancy. “Total” denotes the sum of all cost categories that we consider for our analysis (shown in the top half of the table). “Other” is composed of several smaller cost categories not explicitly shown such as prevention services or special dentist services. The bottom half of the table covers cost categories that we exclude for our analysis.

**FIGURE 3:**
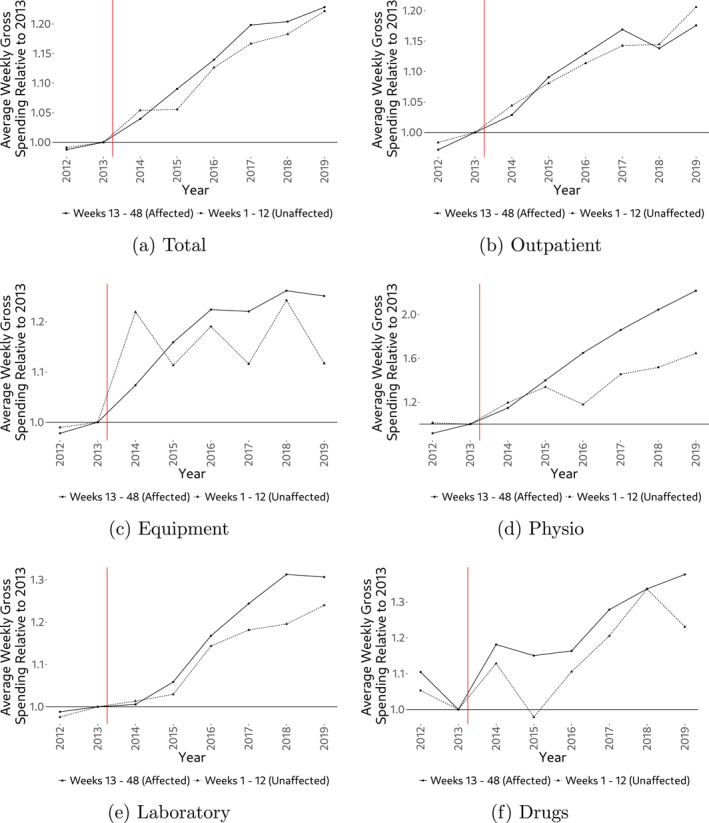
Average weekly spending (2012–2019). The figure shows average weekly spending relative to 2013 for both affected weeks (weeks 13–48, with cost‐sharing exemption) and unaffected pregnancy weeks (weeks 1–12, without cost‐sharing exemption). The vertical red line shows the introduction of the cost‐sharing exemption.

**FIGURE 4:**
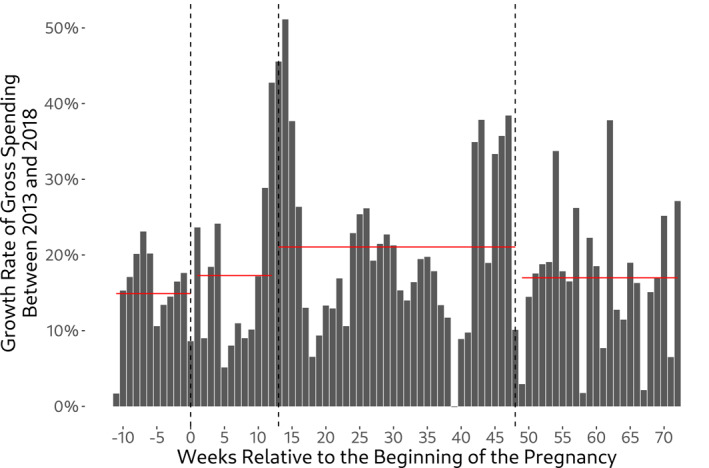
Relative cost growth by pregnancy week between 2013 and 2018. For each pregnancy week, the graph shows the growth rate in health care costs between 2013 and 2018. In addition, red horizontal lines denote average growth rates by group (weeks −11 to 0 refer to the period prior to the beginning of the pregnancy; weeks 1–12 refer to the unaffected group; weeks 13–48 refer to the affected group which has been subject to the cost‐sharing exemption; weeks 49–72 refer to the post‐pregnancy period). Figure based on total gross spending as defined in Table 1.

Table 1 indicates strong growth in gross spending overall. However, there is generally no evidence that relative cost growth in the affected group strongly outpaces the one from the unaffected group. This is easily seen in Figure 3 where we show growth in average gross spending visually by cost category.^11^ The two major exceptions are physiotherapy and laboratory services, for which relative growth in the affected weeks strongly outpaces relative growth in the unaffected weeks. Another way to see the lack of a strong policy effect on aggregate demand is to take a look at pregnancy‐week‐specific cost growth (Figure 4). While we indeed observe a higher average growth rate in aggregate demand for the affected weeks, the difference to both the unaffected weeks and also the post‐pregnancy period is rather small (approx. 2% points). Also note that the figure suggests a somewhat higher growth rate in the immediate vicinity of week 13, which could be indicative of some demand shifting in response to the exemption. Given that we do not observe the exact week of pregnancy, measurement error may partly contribute to the spread of this pattern around the cutoff.

**FIGURE 5:**
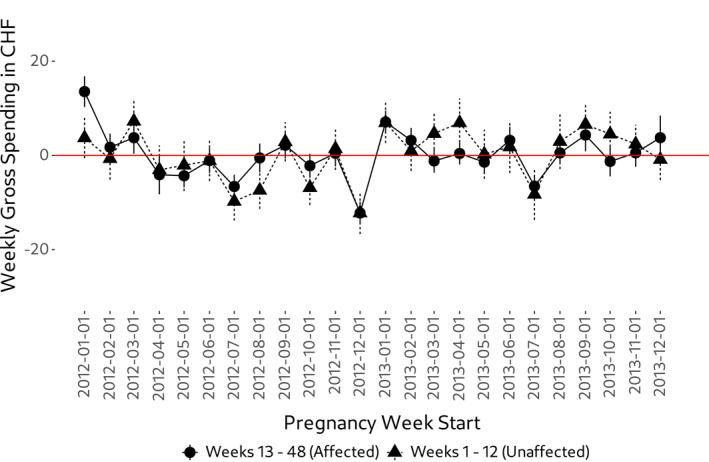
Average gross spending for a pregnancy week relative to 2‐year average, by month. The Figure shows average gross spending among all pregnancy weeks within any given calendar month in the pre‐policy years 2012 and 2013, by group and relative to the group‐specific 2‐year average. Vertical lines denote a 95%‐confidence interval. Assignment of pregnancy weeks to calendar months is based on the first day of each pregnancy week.

A potential concern in our empirical strategy is that the unaffected weeks are subject to different cost trends already prior to the policy. In Figure 5 we show average gross spending for all pregnancy weeks within a calendar month between 2012 and 2013. Using Figure 5 it can easily be recognized that the unaffected and affected group move highly parallel in terms of gross spending prior to the policy introduction. For this reasons, we think that the concern of different pre‐trends is negligible.

## 4 Results

**FIGURE 6:**
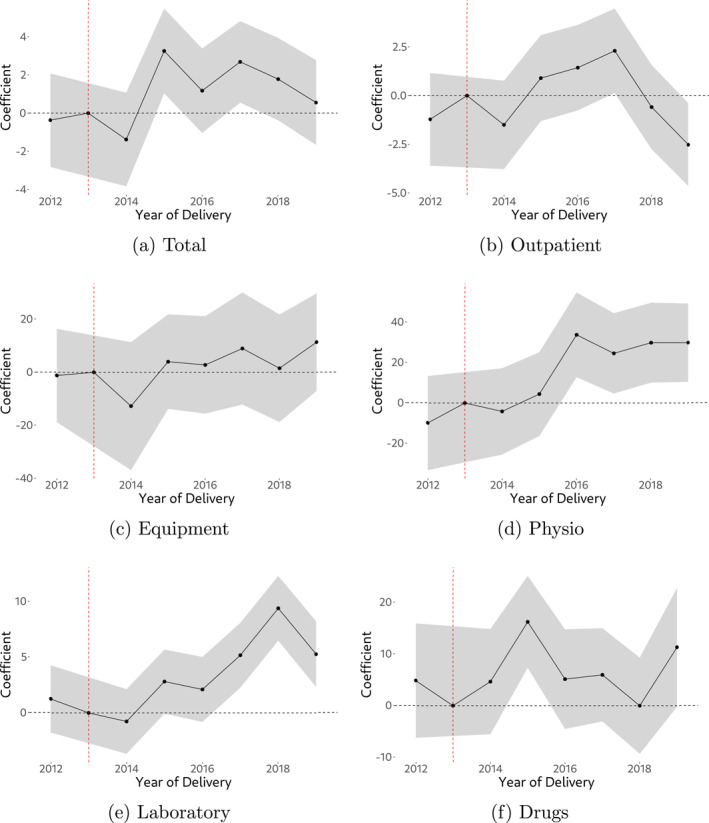
Demand response to the policy change for different cost categories. Regression coefficients (interaction terms) from estimating Equation (1) using the data described in Section 3.2 for different cost categories, including a 95% confidence interval on coefficients (wald confidence intervals, assuming asymptotic normality).

Figure 6 shows interaction terms (our main coefficients of interest from estimating Equation (1)) for various cost categories. Numeric regression output is provided in Appendix Table A2. In line with descriptive analysis, we do not find evidence of persistent policy effects for most cost categories, except for physiotherapy and laboratory services.^12^ For total gross spending, we find positive but fuzzy policy effects. Importantly, we do not find statistically significant differences in pre‐policy cost growth for all cost categories. Focusing on physiotherapy and laboratory services, they exhibit a positive and in most years statistically significant treatment effect after the introduction of the cost‐sharing exemption in 2014. With respect to physiotherapy, we also find large effect sizes indicating that costs have risen by an extra 30% relative to the unaffected group. For physiotherapy, we also see an increase of the treatment effect over time, which seems to stabilize after 2016. In the year prior to 2016, the treatment effect is not statistically different from zero. One potential explanation for this pattern is that (expectant) mothers were not aware of the cost‐sharing exemption in the early years after its implementation.

Given that previous analysis revealed positive but fuzzy policy effects for total spending, we deviate in a next step from our preferred specification in order to increase statistical power and efficiency: First, we included additional control variables (deductible and demographics) into our model. Demographics included age of mother, canton, and language of individuals. Second, we included also observations for which we do not observe the entire pregnancy. The results are shown in Table 2. They are qualitatively very similar to our main results.^13^ As we have more statistical power, however, the standard errors are smaller and some coefficients become statistically significant. In particular, the estimates for total gross spending are now statistically significantly different from zero after 2014. This supports our conclusion, that the policy change had a positive effect on overall demand mainly driven by an effect on the demand for physiotherapy and laboratory services. However, the inclusion of incomplete (not fully observed) pregnancy cases also leads to somewhat larger (absolute) effect sizes, which suggests that pregnant women who switch their health insurer differ slightly in their demand response.^14^ As evident from Table A1 in the Appendix, switching women are also different in terms of observable characteristics. They are 1.2 years younger, have a higher average deductible, and are less often German‐speaking. Although it is very likely that switching behavior is orthogonal to the policy change, we therefore stick to our main specification.

**TABLE 2:** Demand response to the policy change when re‐including incomplete (not fully observed) pregnancy cases and using additional controls.

	Total	Outpatient	Equipment	Physio	Laboratory	Drugs
$\gamma_{2012}$	−0.3	−1.4	−4.1	−14.0	2.0	4.6
	(1.1)	(1.1)	(8.6)	(11.2)	(1.4)	(5.2)
$\gamma_{2014}$	−0.9	−1.5	−13	−5.1	−0.9	4.4
	(1.1)	(1.1)	(11.4)	(10.3)	(1.4)	(4.5)
$\gamma_{2015}$	3.9***	1.3	1.9	2.6	3.2**	17.0***
	(1.0)	(1.0)	(8.7)	(10.1)	(1.3)	(4.1)
$\gamma_{2016}$	1.8*	2.1**	2.4	33.5***	2.0	7.6
	(1.0)	(1.0)	(8.9)	(10.1)	(1.4)	(4.7)
$\gamma_{2017}$	3.6***	3.0***	7.9	23.6**	5.6***	7.3
	(1.0)	(1.0)	(10.2)	(9.6)	(1.4)	(4.5)
$\gamma_{2018}$	2.5**	−0.1	−0.3	29.3***	9.4***	2.5
	(1.0)	(1.0)	(9.7)	(9.5)	(1.4)	(4.6)
$\gamma_{2019}$	2.2**	−1.3	7.5	27.1***	6.5***	13.7**
	(1.0)	(1.0)	(9.8)	(9.3)	(1.4)	(5.5)
Obs. (In thousands)	4921	4921	4921	4921	4921	4921
Individuals	107,524	107,524	107,524	107,524	107,524	107,524

*Note:* The table shows differences in growth rates between the affected pregnancy weeks (weeks 13 until 8th week after delivery) and the unaffected pregnancy weeks (weeks 1–12) for various cost categories. Numbers are estimated using the generalized linear model described in Section 3.1 and can be interpreted as percentage points. These coefficients denote the causal response by expectant mothers to the cost‐sharing exemption. The estimated coefficients as well as standard errors (in parenthesis) are multiplied by 100 and rounded to 1 digit. The generalized linear regression in this case has been estimated using both, fully‐observed and partially‐observed pregnancy cases as described in Section 3.2 as well as using additional controls. Added controls include the deductible, age of mother, canton, and language of individuals. For results without these adjustments, we refer to Table A2 in the Appendix.

*** significant at the 1% level.

** significant at the 5% level.

* significant at the 10% level.

We conduct several robustness checks to examine possible patterns of bunching and timing of care (demand shifting). Specifically, to account for potential bunching around pregnancy week 13 and possible shifting of care from weeks 1–12 into later pregnancy weeks, we exclude weeks 11–14 from the data (Table A5 in the Appendix). The results remain qualitatively similar, with slightly stronger effect sizes for total gross spending. These findings are robust to further exclusions: dropping weeks 6–8 after delivery (to account for possible bunching around the eighth postpartum week, see Appendix Table A6) and dropping weeks 15 and 16 (Appendix Table A7). While the primary estimates of interest remain qualitatively unchanged in all of these specifications, we observe that some pre‐treatment coefficients, including those for the year 2014, are affected by these exclusions in terms of size, sign and statistical significance. This may suggest some degree of demand shifting. Therefore, we cannot fully rule out the possibility that part of the observed demand response reflects changes in the timing of care.

We also conduct robustness checks related to the econometric specification. We use an alternative control group—the 12 weeks prior to the start of pregnancy—instead of the first 12 weeks of pregnancy, to address concerns that our fixed pregnancy length assumption might misassign treatments. The results from this specification remain highly consistent with the baseline findings (Appendix Table A8). In addition, we verify that our results are not sensitive to the inclusion or exclusion of pregnancy week fixed effects, which makes no difference to our estimates (Appendix Table A9). Furthermore, although inpatient care was excluded from the main analysis, we include it in a robustness regression. Although the estimates do not provide strong evidence of a policy effect on inpatient care (Appendix Table A10), the presence of significant pre‐trends and low precision of the estimates limit the strength of this conclusion. Lastly, we estimate a simplified 2 × 2 difference‐in‐differences model, comparing cost trends in the affected weeks before and after the policy introduction relative to unaffected weeks, using a single *Affected* × *Post* interaction term (Appendix Table A11). The interaction is highly significant for total spending, physiotherapy, and laboratory services, with estimated spending growth higher by approximately 2, 30, and 4.5% points, respectively. These estimates are broadly in line with the average effects found in our main specification.

### 4.1 Heterogeneity Analysis by Income

Our estimates suggest that the cost‐sharing exemption had a positive although relatively small effect on overall healthcare use. One potential explanation for this finding is that health insurance coverage in Switzerland has already been comprehensive prior to the introduction of the cost‐sharing exemption and Swiss citizens are among the most wealthy in the world. Based on Figure 1 the individual savings due to the cost‐sharing exemption are somewhere between CHF 10 and 25 per week, or between CHF 40 and 100 per month.^15^ Therefore, the price reduction for health care due to the cost‐sharing exemption might not be large enough for major parts of the population to induce a visible amount of extra demand for care. However, individual savings matter more for people with low incomes. Hence, we conduct a heterogeneity analysis by income using a binary setup (individuals with above‐vs. below‐median income).

**FIGURE 7:**
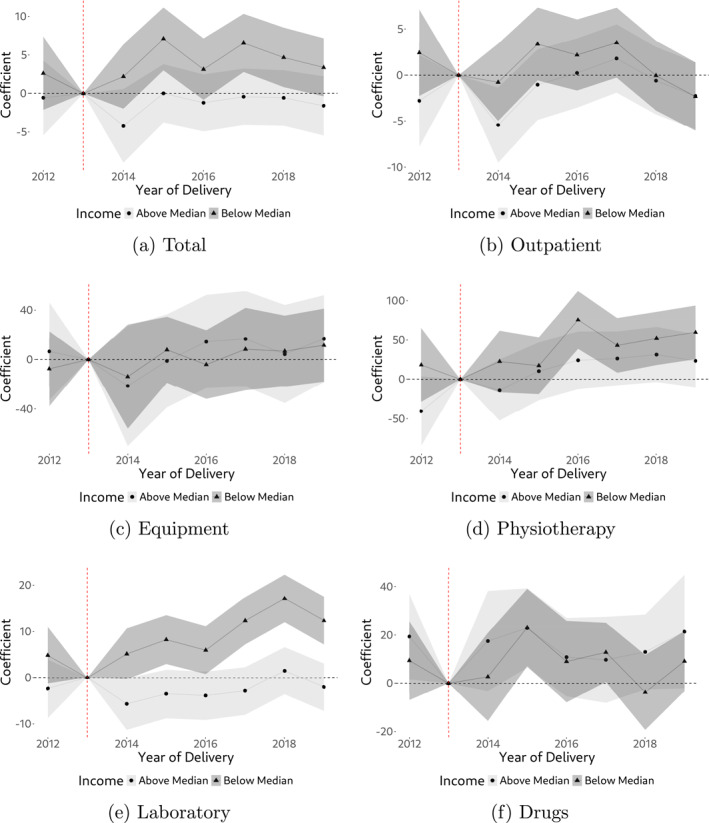
Annual treatment effects by income. The figure shows regression coefficients (interaction terms) from estimating Equation (1) by income, using the data described in Section 3.2, including a 95% confidence interval (Wald confidence intervals).

We find strong heterogeneity in demand responses to the policy by income: While we do not find statistically significant demand responses among above‐median income individuals (including for physiotherapy, although effect sizes still suggest a policy effect), we find clear and statistically significant evidence of a demand response among below‐median income individuals for physiotherapy and laboratory services. Note that effect sizes are high (around 50% for physiotherapy, on average) and around 11% for laboratory services (on average). In addition, we now also find quite consistent evidence of a policy effect on total gross spending. Our findings are shown visually in Figure 7. An important observation in these Figures is that the demand response among below‐median income individuals sets in immediately after the policy came into effect. This is something that the overall effects (Figure 6 and Appendix Table A2) have not been able to indicate as clearly. For all other outcomes (outpatient services, equipment, drugs) we continue to have no evidence of a persistent policy effect, even among below‐median income individuals.

### 4.2 Health Outcomes

So far, our analysis centered around the policy's impact on demand, and our key finding was a positive but rather small overall demand response with important heterogeneity with respect to some categories of services as well as by income. An important complementary question concerns the policy's impact on health. On the one hand, the extra demand induced might be beneficial for the child. On the other hand, the reduction of cost sharing reduces financial burden and might lead to health benefits for the mother.

**FIGURE 8:**
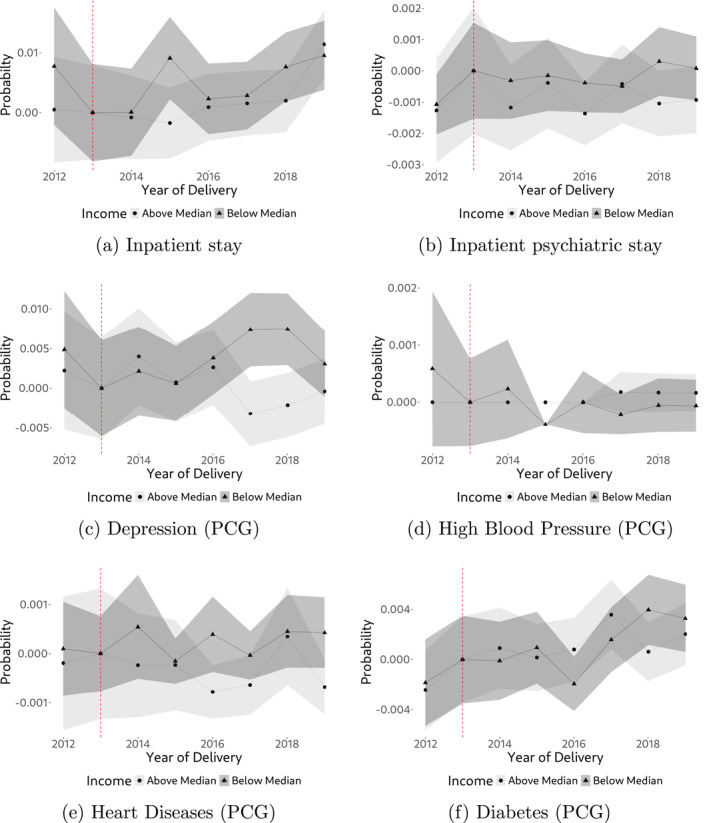
Maternal health outcomes, by income. The figure shows trends in different maternal health outcomes, by income. (a) Probability of inpatient stay of the mother in the post‐pregnancy period. (b) Probability of inpatient psychiatric stay of the mother in the post‐pregnancy period. (c)–(f) Probability of the mother having a chronic diseases during pregnancy and in the post pregnancy period, using pharmaceutical cost groups (PCG) as indicators of such diseases. Probabilities in all Figures are expressed relative to 2013.

In general, the type of (Swiss) health insurance data to which we have access is not very rich in health information, such as diagnosis. However, we have access to some specific health indicators that we can use for analysis. First and concerning the mother, we evaluate changes in post‐pregnancy inpatient and post‐pregnancy psychiatric inpatient stay probability. If the policy substantially reduces stress, we might see a reduction in psychiatric stays, for example. Furthermore, regarding maternal health, we examine the probability of mothers having specific pharmaceutical cost groups (PCGs) during and after the pregnancy period. PCGs are drug‐based indicators of chronic diseases, meaning they serve as markers for both the diagnosis and effective treatment of a condition. An increase in PCG measures can therefore indicate either a decline in health or improved management of an existing condition. We specifically focus on PCGs related to depression, heart diseases, high blood pressure, and diabetes. For each of these conditions, we assess changes in the empirical average over time, comparing individuals with below‐median and above‐median incomes. The results, presented in Figure 8, show no consistent evidence of a policy impact on maternal health, with the exception of 2 years in which significant differences in depression rates are observed. However, as these differences emerge several years after the reform and do not persist, we are skeptical that they reflect health effects directly attributable to the policy. Overall, we find no evidence of a policy effect on maternal health. However, this does not necessarily imply that the reform had no impact, as it may have affected other health outcomes that we are not able to observe.

**FIGURE 9:**
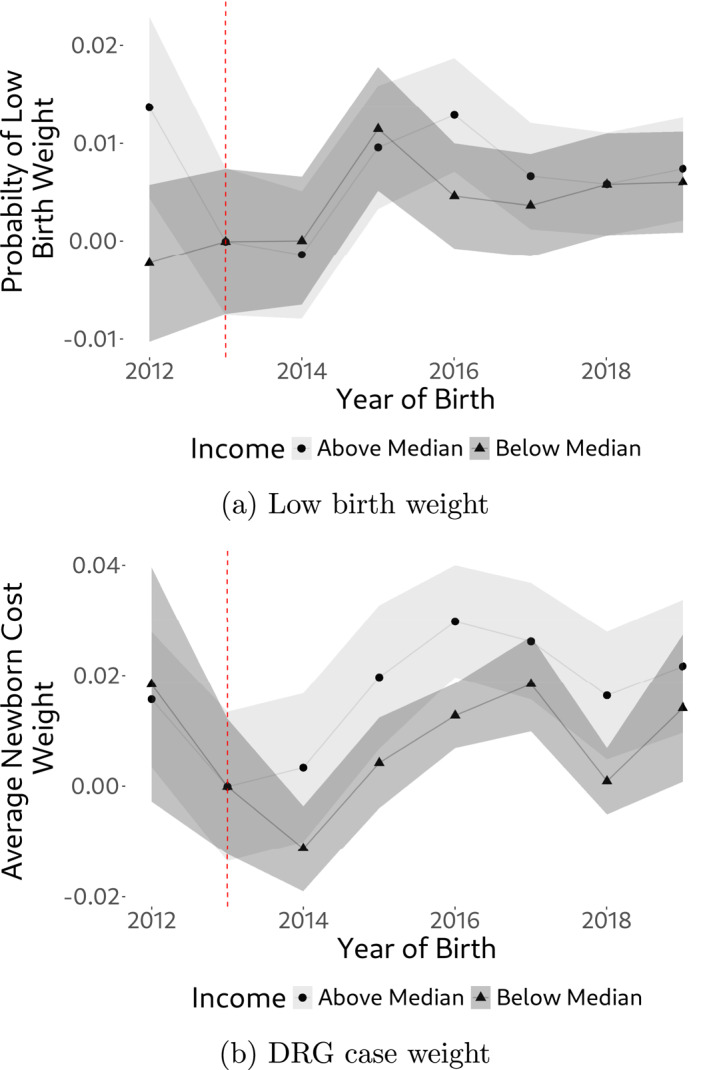
Health indicators of newborns, by income. The figure shows trends in health indicators of newborns, by income. (a) Probability of low birth weight of the newborn. (b) Average DRG case weight of newborns. Metrics in all Figures are expressed relative to 2013.

Next, and concerning the child, we have access to a binary low‐birth weight indicator (< 2,500 g) which serves as a crude measurement of health at birth. In addition, we also have access to the newborn's DRG case weight. The DRG case weight is a relative measure of patient morbidity (see, e.g., Hochuli 2020) and therefore, in the context of newborns, provides a comprehensive, single‐number health assessment of the child at birth (the less healthy a child at birth, the higher the case weight). Results are shown in Figure 9. In this case, we do find some suggestive evidence of a positive policy impact on newborn's health when considering the DRG case weight statistics. While being almost identical prior to policy introduction, there is a constant case weight gap of approx. 0.02 between below‐median and above‐median income individuals after the policy change. Given that a lower case weight implies better health, this gap corresponds to an approximate health gain of 7% of the below‐median income individuals relative to above‐median income individuals. Interestingly, and consistent with the demand response in Figure 7, the health gap starts to set in almost immediately following the onset of the policy. This suggestive finding of improved newborn health aligns with Epure et al. (2023), who report positive health effects of the reform, specifically in terms of birth weight.^16^

## 5 Conclusion

When health insurance coverage is expanded or cost‐sharing is reduced, health care becomes less expensive for insured individuals. On the one hand, this can have positive health effects as more health care is consumed. In the particular case of pregnancies, which is the focus of our research, an increased use of prenatal care is widely believed to improve infant health (Conway and Deb 2005). On the other hand, health care consumption could be inefficiently high without cost sharing. We analyze a cost‐sharing exemption for (expectant) mothers in Switzerland, which was introduced on March 1st in 2014 and which has made all care free between the 13th week of pregnancy and 8 weeks after delivery.

Besides annually shifting health care costs of 40–50 million Swiss Francs from the (expectant) mothers to the health insurer, the cost‐sharing exemption also lead to a demand response, primarily among below‐median income individuals. In this group, health care spending increased by roughly 150 Swiss Francs per pregnancy resulting in additional annual expenditures of 6‒7 million Swiss Francs. Given that primarily below‐median income individuals benefited from the policy, we conclude that the cost‐sharing exemption increased equity among pregnant women. In addition, we find suggestive evidence that this contributed to an improvement in newborn health among below‐median income individuals. This highlights that there is not only a trade‐off between welfare gains from risk protection and welfare losses from moral hazard, but also between health care costs, health, and equity.

Our research has some limitations. First, it relies on the assumption of a standardized pregnancy length, as we do not observe the actual start of a pregnancy. This may lead to some misallocation of cost data across pregnancy weeks. However, our various robustness checks—particularly the use of an alternative control group and the exclusion of pregnancy weeks around the 13th week—give us confidence that this data imprecision does not materially affect our analysis. A related concern is that the reform itself could plausibly influence gestation length, for example, by reducing pregnancy complications, which could introduce bias into our analysis. However, a recent study by Epure et al. (2023), which examines the same reform, finds only a negligible effect on gestation length, mitigating this concern. Second, and although our primary estimates remain similar across various robustness checks, we cannot rule out that some of the demand response we measure is due to demand shifting from weeks 1–12 into weeks 13 and later. However, irrespective of whether the demand represents shifted or additional care, the financial burden borne by the general public rises. Moreover, the observed improvement in newborn health among below‐median income individuals suggests that at least some of the demand response corresponds to an actual increase in health care utilization. Third, our assessment of the health impact of the policy is clearly incomplete. While we consider our demand analysis to be well‐suited for a policy assessment on demand, we do not have the same confidence when it comes to our health assessment. If more detailed data were available (such as diagnosis), this issue could be tackled.

## Disclosure

We are grateful to seminar and conference participants in Bern (Switzerland), Halle (Saale, Germany), and Vienna (Austria) for their valuable comments. All remaining errors are our own.

## Conflicts of Interest

Philip Hochuli reports Personal fees from CSS, outside the submitted work. Dr. Schmid reports Personal fees from CSS Institute, outside the submitted work.

## Figures and Tables

**FIGURE A1:**
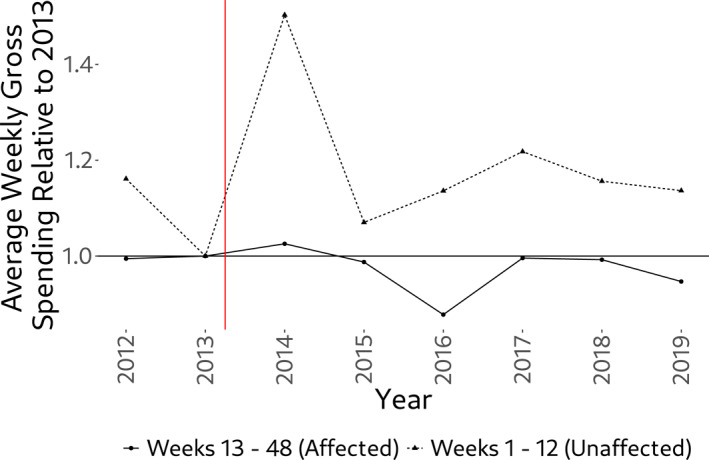
Average weekly spending on other services (2012–2019). The figure shows average weekly spending for “other” services relative to 2013 for both affected weeks (weeks 13–48, with cost‐sharing exemption) and unaffected pregnancy weeks (weeks 1–12, without cost‐sharing exemption). For the definition of “other” services, see Table 1. The vertical red line shows the introduction of the cost‐sharing exemption.

## Data Availability

The data that support the findings of this study are available from CSS. Restrictions apply to the availability of these data, which were used under license for this study. Data are available from the author(s) with the permission of CSS. Data recipients have to declare to comply with Swiss data protection laws.

## Endnotes

^1^
Interestingly, the federal parliament expressed not to expect any significant impact on demand from the policy change and, in consequence, no impact on health. It was aware, however, that the cost‐sharing exemption would shift health care costs from the (expectant) mothers to the general public via insurance. The additional costs for the general public were estimated at 33.6 mio. Swiss francs per year.

^2^
A few pregnancy‐related services were already exempt from cost‐sharing, namely a limited number of check‐ups provided by doctors and midwives during and after pregnancy, the delivery at home, in a hospital or in a birth center, prenatal classes, breastfeeding counseling, and midwife services before, during and after childbirth.

^3^
For children, the individual deductible in the standard health plan is zero. The voluntary deductible ranges from CHF 100 to 600; children can also opt for health plans with managed care features. Irrespective of health plan choice, the individual stop‐loss amount is CHF 350 or, if a family has more than two children, cumulatively CHF 700 for all children.

^4^
These services are also covered in case of accident. However, all individuals with more than 8 hours of paid work per week are covered by employer sponsored accident insurance.

^5^
In addition, some services provided under a cantonal prevention program are also exempt from the deductible (e.g., breast cancer screening, colon cancer screening, and flu vaccinations).

^6^
Distinguishing between maternity‐related treatments and other care was considered difficult and not feasible.

^7^
Because our data does not comprise the starting date of the 13th week of pregnancy, we have to impute it. Hence, some weeks with actual cost‐sharing exemption are assigned to the pre‐exemption weeks 1–12; similarly, some weeks with cost‐sharing are assigned to weeks with cost‐sharing exemption in place. As a result, cost‐sharing of affected weeks does not drop precisely to zero and cost‐sharing in unaffected weeks exhibit a slight decline in the graph.

^8^
To estimate the gestation length, we use more recent data, which contains also information on the start day of the 13th pregnancy week determined by the expectant mother's physician. Given this date and the date of childbirth, we calculate the median gestation length. Note that we also tried a machine‐learning‐based approach in which we inferred the pregnancy length using an ML‐model being trained on this more recent data. We found, however, that this approach did not perform better than the simple method, and we therefore prefer the simple approach with a standardized pregnancy length.

^9^
Market share is based on the risk equalization statistic, which provides the number of women in age groups 0–18, 19–25, 26–30, 31–35, and so on.

^10^
Number of insured as of December 31, 2012: 1,213,204. Number of insured as of Dec 12, 2020: 1,398,041 (+15.2%).

^11^
For the “other” cost category, we refer to Appendix Figure A1.

^12^
In addition to physiotherapy and laboratory services, drug costs show a positive significant effect in two separate years. However, there is no clear pattern, and the statistical significance appears to be driven more by the varying size of the coefficients rather than a change in behavior. Therefore, we refrain from interpreting these effects as a consequence of the policy change.

^13^
Tables A3 and A4 in the Appendix show the results separately for the inclusion of additional controls and the inclusion of incomplete pregnancy cases, respectively.

^14^
Including additional controls only does not impact the effect size, which implies that the treatment is not correlated with these observed factors.

^15^
For comparison, a median monthly salary in 2020 was roughly 6600 Swiss francs in Switzerland (before taxes).

^16^
It is worth noting that while Epure et al. (2023) find positive effects among children of parents not at risk of poverty, our analysis identifies these effects within the below‐median income group. However, this is not contradictory, as there is significant overlap between parents not at risk of poverty and those with below‐median income.

## Appendix A

### Additional Results

**TABLE A1:** Descriptive comparison of incomplete pregnancy cases versus complete pregnancy cases.

Incomplete	Obs.	Cases	Age	Deductible	Weekly spending	German‐speaking
0	7,257t	86,388	31.9	1196.1	75.0	67%
1	1,266t	21,167	30.7	1257.9	77.1	62%

*Note:* Descriptive comparison of fully‐observed pregnancy cases (incomplete = 0) and partially‐observed cases. Numbers denote empirical averages and are rounded to 1 digit. Weekly spending is denoted in CHF.

**TABLE A2:** Demand response to the policy change for different cost categories.

	Total	Outpatient	Equipment	Physio	Laboratory	Drugs
$\gamma_{2012}$	−0.4	−1.2	−1.2	−9.9	1.2	4.8
	(1.2)	(1.2)	(9.0)	(11.8)	(1.5)	(5.6)
$\gamma_{2014}$	−1.4	−1.5	−12.7	−4.1	−0.8	4.6
	(1.2)	(1.2)	(12.3)	(10.9)	(1.5)	(5.2)
$\gamma_{2015}$	3.2***	0.9	4.0	4.4	2.8*	16.2***
	(1.1)	(1.1)	(9.1)	(10.5)	(1.5)	(4.5)
$\gamma_{2016}$	1.2	1.4	2.8	33.7***	2.1	5.1
	(1.1)	(1.1)	(9.3)	(10.7)	(1.5)	(4.9)
$\gamma_{2017}$	2.7**	2.3**	8.9	24.5**	5.2***	5.9
	(1.1)	(1.1)	(10.7)	(10.1)	(1.5)	(4.6)
$\gamma_{2018}$	1.8	−0.6	1.5	29.8***	9.4***	0.0
	(1.1)	(1.1)	(10.3)	(10.1)	(1.5)	(4.7)
$\gamma_{2019}$	0.6	−2.5**	11.3	29.8***	5.2***	11.3*
	(1.1)	(1.1)	(9.3)	(9.9)	(1.5)	(5.8)
Obs. (in thousands)	4147	4147	4147	4147	4147	4147
Individuals	86,388	86,388	86,388	86,388	86,388	86,388

*Note:* The table shows differences in growth rates between the affected pregnancy weeks (weeks 13 until 8th week after delivery) and the unaffected pregnancy weeks (weeks 1–12) for various cost categories. Numbers are estimated using the generalized linear model described in Section 3.1 and can be interpreted as percentage points. These coefficients denote the causal response by expectant mothers to the cost‐sharing exemption. The estimated coefficients as well as standard errors (in parenthesis) are multiplied by 100 and rounded to 1 digit.

*** significant at the 1% level.

** significant at the 5% level.

* significant at the 10% level.

**TABLE A3:** Demand response to the policy change for different cost categories when including additional control variables.

	Total	Outpatient	Equipment	Physio	Laboratory	Drugs
$\gamma_{2012}$	−0.3	−1.2	−1.2	−9.8	1.3	4.9
	(1.2)	(1.2)	(9.0)	(11.8)	(1.5)	(5.6)
$\gamma_{2014}$	−1.4	−1.5	−12.7	−4.2	−0.8	4.6
	(1.2)	(1.2)	(12.3)	(10.9)	(1.5)	(5.2)
$\gamma_{2015}$	3.4***	1.0	4.2	4.7	2.9**	16.6***
	(1.1)	(1.1)	(9.1)	(10.5)	(1.5)	(4.5)
$\gamma_{2016}$	1.4	1.6	3.1	34.1***	2.2	5.5
	(1.1)	(1.1)	(9.3)	(10.7)	(1.5)	(4.9)
$\gamma_{2017}$	3.0***	2.6**	9.4	25.1**	5.3***	6.8
	(1.1)	(1.1)	(10.7)	(10.1)	(1.5)	(4.6)
$\gamma_{2018}$	2.1*	−0.3	1.9	30.5***	9.6***	0.8
	(1.1)	(1.1)	(10.3)	(10.1)	(1.5)	(4.7)
$\gamma_{2019}$	0.9	−2.3**	11.7	30.4***	5.4***	11.9**
	(1.1)	(1.1)	(9.3)	(9.9)	(1.5)	(5.9)
Obs. (in thousands)	4147	4147	4147	4147	4147	4147
Individuals	86,388	86,388	86,388	86,388	86,388	86,388

*Note:* Interpretation identical to Table A2 with the difference that the generalized linear regression includes additional controls. Added controls include the deductible, age of mother, canton, and language of individuals.

*** significant at the 1% level.

** significant at the 5% level.

* significant at the 10% level.

**TABLE A4:** Demand response to the policy change for different cost categories when re‐including incomplete (not fully observed) pregnancy cases.

	Total	Outpatient	Equipment	Physio	Laboratory	Drugs
$\gamma_{2012}$	−0.5	−1.6	−4.2	−14.5	1.8	4.4
	(1.1)	(1.1)	(8.6)	(11.2)	(1.4)	(5.2)
$\gamma_{2014}$	−0.8	−1.4	−12.9	−5.1	−0.9	4.6
	(1.1)	(1.1)	(11.4)	(10.3)	(1.4)	(4.5)
$\gamma_{2015}$	3.6***	0.9	1.8	1.9	2.8**	16.5***
	(1.0)	(1.0)	(8.7)	(10.1)	(1.3)	(4.0)
$\gamma_{2016}$	1.3	1.5	2.0	32.4***	1.6	6.8
	(1.0)	(1.0)	(8.9)	(10.1)	(1.4)	(4.7)
$\gamma_{2017}$	3.1***	2.6**	7.5	22.7**	5.3***	6.3
	(1.0)	(1.0)	(10.2)	(9.6)	(1.4)	(4.5)
$\gamma_{2018}$	2.0**	−0.5	−0.8	28.2***	9.0***	1.5
	(1.0)	(1.0)	(9.7)	(9.5)	(1.4)	(4.6)
$\gamma_{2019}$	1.8*	−1.6	7.2	26.1***	6.2***	13.1**
	(1.0)	(1.0)	(9.8)	(9.3)	(1.4)	(5.5)
Obs. (in thousands)	4921	4921	4921	4921	4921	4921
Individuals	107,524	107,524	107,524	107,524	107,524	107,524

*Note:* Interpretation identical to Table A2. The generalized linear regression in this case has been estimated using both, fully‐observed and partially‐observed pregnancy cases as described in Section 3.2.

*** significant at the 1% level.

** significant at the 5% level.

* significant at the 10% level.

**TABLE A5:** Demand response to the policy change for different cost categories when dropping weeks 11–14.

	Total	Outpatient	Equipment	Physio	Laboratory	Drugs
$\gamma_{2012}$	−1.0	−2.2	4.4	−12.3	0.7	3.6
	(1.4)	(1.4)	(12.4)	(13.0)	(1.7)	(5.8)
$\gamma_{2014}$	−2.7**	−2.3*	−18.8	−14.0	−2.3	2.5
	(1.3)	(1.3)	(17.1)	(11.8)	(1.6)	(4.6)
$\gamma_{2015}$	2.8**	0.2	9.2	3.0	2.4	14.6***
	(1.3)	(1.3)	(12.6)	(11.6)	(1.6)	(4.3)
$\gamma_{2016}$	2.8**	0.9	6.3	28.9**	5.1***	2.6
	(1.3)	(1.3)	(13.0)	(11.7)	(1.6)	(5.7)
$\gamma_{2017}$	3.9***	1.0	11.6	18.3*	9.1***	3.3
	(1.3)	(1.3)	(15.6)	(11.1)	(1.6)	(5.6)
$\gamma_{2018}$	3.7***	−2.3*	−2.8	31.8***	15.5***	−0.8
	(1.3)	(1.3)	(14.2)	(11.1)	(1.6)	(5.0)
$\gamma_{2019}$	3.9***	−3.9***	9.5	26.5**	13.3***	15.2***
	(1.3)	(1.3)	(13.3)	(10.8)	(1.7)	(4.8)
Obs. (in thousands)	3801	3801	3801	3801	3801	3801
Individuals	86,388	86,388	86,388	86,388	86,388	86,388

*Note:* Interpretation identical to Table A2 with the exception that pregnancy weeks 11–14 have been dropped from the data.

*** significant at the 1% level.

** significant at the 5% level.

* significant at the 10% level.

**TABLE A6:** Demand response to the policy change for different cost categories when dropping weeks 11–14 as well as post‐delivery weeks 6–8.

	Total	Outpatient	Equipment	Physio	Laboratory	Drugs
$\gamma_{2012}$	−1.3	−2.2	4.3	−11.1	0.1	2.6
	(1.4)	(1.4)	(12.4)	(13.1)	(1.7)	(6.0)
$\gamma_{2014}$	−3.0**	−2.6*	−19.4	−13.1	−1.8	1.7
	(1.3)	(1.4)	(17.1)	(11.9)	(1.7)	(4.5)
$\gamma_{2015}$	2.3*	−0.4	8.7	3.8	3.0*	13.7***
	(1.3)	(1.3)	(12.6)	(11.7)	(1.7)	(4.2)
$\gamma_{2016}$	2.4*	0.5	5.5	29.3**	5.6***	1.9
	(1.3)	(1.3)	(13.0)	(11.8)	(1.6)	(5.6)
$\gamma_{2017}$	3.5***	0.6	10.5	19.0*	9.8***	2.6
	(1.3)	(1.3)	(15.7)	(11.2)	(1.7)	(5.8)
$\gamma_{2018}$	2.9**	−3.5***	−3.1	32.2***	16.0***	−1.7
	(1.3)	(1.3)	(14.2)	(11.2)	(1.7)	(5.2)
$\gamma_{2019}$	3.1**	−4.9***	8.1	25.1**	13.7***	15.6***
	(1.3)	(1.3)	(13.2)	(10.9)	(1.7)	(4.9)
Obs. (in thousands)	3542	3542	3542	3542	3542	3542
Individuals	86,388	86,388	86,388	86,388	86,388	86,388

*Note:* Interpretation identical to Table A2 with the exception that pregnancy weeks 11–14 as well as post‐delivery weeks 6–8 have been dropped from the data.

*** significant at the 1% level.

** significant at the 5% level.

* significant at the 10% level.

**TABLE A7:** Demand response to the policy change for different cost categories when dropping weeks 11–16.

	Total	Outpatient	Equipment	Physio	Laboratory	Drugs
$\gamma_{2012}$	−1.0	−2.1	4.8	−11.5	0.8	4.2
	(1.4)	(1.4)	(12.4)	(13.1)	(1.7)	(6.0)
$\gamma_{2014}$	−2.7**	−2.4*	−18.4	−13.5	−1.8	2.8
	(1.3)	(1.4)	(17.1)	(11.9)	(1.6)	(4.5)
$\gamma_{2015}$	2.9**	0.3	9.7	4.2	2.6	15.5***
	(1.3)	(1.3)	(12.6)	(11.7)	(1.6)	(4.4)
$\gamma_{2016}$	2.2*	0.9	6.0	30.0**	2.0	3.1
	(1.3)	(1.3)	(13.0)	(11.8)	(1.6)	(5.7)
$\gamma_{2017}$	3.5***	1.1	11.9	19.1*	6.4***	3.6
	(1.3)	(1.3)	(15.6)	(11.2)	(1.6)	(5.9)
$\gamma_{2018}$	2.9**	−2.5*	−2.9	33.5***	12.2***	−0.6
	(1.3)	(1.3)	(14.2)	(11.2)	(1.6)	(5.1)
$\gamma_{2019}$	3.3**	−4.1***	9.4	27.5**	11.0***	16.1***
	(1.3)	(1.3)	(13.3)	(10.9)	(1.7)	(4.9)
Obs. (in thousands)	3628	3628	3628	3628	3628	3628
Individuals	86,388	86,388	86,388	86,388	86,388	86,388

*Note:* Interpretation identical to Table A2 with the exception that pregnancy weeks 11–16 have been dropped from the data.

*** significant at the 1% level.

** significant at the 5% level.

* significant at the 10% level.

**TABLE A8:** Demand response to the policy change for different cost categories when using weeks before the beginning of a pregnancy as the unaffected group.

	Total	Outpatient	Equipment	Physio	Laboratory	Drugs
$\gamma_{2012}$	−0.4	1.9	17.2	1.1	−4	6.5
	(3.1)	(2.8)	(18.7)	(11.4)	(3.7)	(6.8)
$\gamma_{2014}$	−0.5	−2	9.0	12.7	4.2	5.1
	(2.8)	(2.7)	(16.7)	(10.8)	(3.6)	(7.4)
$\gamma_{2015}$	2.7	−0.7	5.2	17.8*	6.1*	17.2***
	(2.7)	(2.7)	(16.8)	(10.4)	(3.5)	(5.6)
$\gamma_{2016}$	2.1	−0.9	4.8	32.8***	5.0	10.3
	(2.7)	(2.6)	(17.5)	(10.5)	(3.5)	(6.5)
$\gamma_{2017}$	−0.3	−2.7	−10	28.5***	5.8*	5.6
	(2.9)	(2.6)	(21.6)	(10.0)	(3.4)	(6.7)
$\gamma_{2018}$	1.0	−4.1	−1.8	39.8***	10.5***	8.1
	(2.8)	(2.6)	(17.7)	(10.0)	(3.5)	(6.6)
$\gamma_{2019}$	3.5	−1.4	14.6	32.4***	5.6	18.7***
	(2.6)	(2.5)	(19.3)	(9.7)	(3.4)	(6.5)
Obs. (in thousands)	4147	4147	4147	4147	4147	4147
Individuals	86,388	86,388	86,388	86,388	86,388	86,388

*Note:* Interpretation identical to Table A2 with the difference that coefficients denote differences in growth rates between the affected pregnancy weeks and weeks prior to the beginning of the pregnancy (instead of pregnancy weeks 1–12).

*** significant at the 1% level.

** significant at the 5% level.

* significant at the 10% level.

**TABLE A9:** Demand response to the policy change for different cost categories when including pregnancy week fixed effects.

	Total	Outpatient	Equipment	Physio	Laboratory	Drugs
$\gamma_{2012}$	−0.4	−1.2	−1.2	−9.9	1.2	4.8
	(1.2)	(1.2)	(9.0)	(11.8)	(1.5)	(5.6)
$\gamma_{2014}$	−1.4	−1.5	−12.7	−4.1	−0.8	4.6
	(1.2)	(1.2)	(12.3)	(10.9)	(1.5)	(5.2)
$\gamma_{2015}$	3.2***	0.9	4.0	4.4	2.8*	16.2***
	(1.1)	(1.1)	(9.1)	(10.5)	(1.5)	(4.5)
$\gamma_{2016}$	1.2	1.4	2.8	33.7***	2.1	5.1
	(1.1)	(1.1)	(9.3)	(10.7)	(1.5)	(4.9)
$\gamma_{2017}$	2.7**	2.3**	8.9	24.5**	5.2***	5.9
	(1.1)	(1.1)	(10.7)	(10.1)	(1.5)	(4.6)
$\gamma_{2018}$	1.8	−0.6	1.5	29.8***	9.4***	0.0
	(1.1)	(1.1)	(10.3)	(10.1)	(1.5)	(4.7)
$\gamma_{2019}$	0.6	−2.5**	11.3	29.8***	5.2***	11.3*
	(1.1)	(1.1)	(9.3)	(9.9)	(1.5)	(5.8)
Obs. (in thousands)	4147	4147	4147	4147	4147	4147
Individuals	86,388	86,388	86,388	86,388	86,388	86,388

*Note:* Interpretation identical to Table A2 with the exception that the model now includes fixed effects for each pregnancy week.

*** significant at the 1% level.

** significant at the 5% level.

* significant at the 10% level.

**TABLE A10:** Demand response to the policy change for inpatient care.

	Estimate
$\gamma_{2012}$	−22.4*
	(12.9)
$\gamma_{2014}$	−11.3
	(13.1)
$\gamma_{2015}$	1.4
	(12.9)
$\gamma_{2016}$	−7.8
	(12.3)
$\gamma_{2017}$	3.8
	(12.6)
$\gamma_{2018}$	2.4
	(13.0)
$\gamma_{2019}$	−3.0
	(12.6)
Obs. (in thousands)	4147
Individuals	86,388

*Note:* Interpretation identical to Table A2.

* significant at the 10% level.

**TABLE A11:** Demand response to the policy change for different cost categories when collapsing our main specification into a simple 2 × 2 period difference‐in‐differences model.

	Total	Outpatient	Equipment	Physio	Laboratory	Drugs
Intercept	446.1***	375.8***	−43.4***	−37.6***	349.7***	191.0***
	(0.6)	(0.6)	(5.2)	(5.8)	(0.6)	(2.9)
Affected	11.3***	29.0***	157.2***	59.1***	−41.2***	27.6***
	(0.6)	(0.6)	(4.5)	(5.9)	(0.8)	(2.8)
Post	14.7***	13.9***	15.0**	35.5***	16.1***	13.7***
	(0.8)	(0.7)	(6.3)	(6.4)	(0.7)	(4.6)
Affected×Post	2.0***	0.8	6.3	30.1***	4.5***	4.9
	(0.7)	(0.7)	(5.5)	(6.5)	(0.9)	(3.4)
Obs. (in thousands)	3669	3669	3669	3669	3669	3669
Individuals	76,445	76,445	76,445	76,445	76,445	76,445

*Note:* We estimate the same model as described in Section 3 with the difference that instead of estimating year‐specific effects, we categorize years into pre‐policy years (2012–2013) and post‐policy years (2015–2019). Year 2014 has been excluded from the data. We take pre‐policy years as reference years. The coefficients Affected×Post are the coefficients of primary interest. They denote the relative cost growth of the affected weeks relative to the unaffected weeks. The estimated coefficients as well as standard errors (in parenthesis) are multiplied by 100 and rounded to 1 digit.

*** significant at the 1% level.

** significant at the 5% level.
